# The Spectrum of Cancers in West Africa: Associations with Human Immunodeficiency Virus

**DOI:** 10.1371/journal.pone.0048108

**Published:** 2012-10-29

**Authors:** Aristophane Tanon, Antoine Jaquet, Didier K. Ekouevi, Jocelyn Akakpo, Innocent Adoubi, Isidore Diomande, Fabien Houngbe, Marcel D. Zannou, Annie J. Sasco, Serge P. Eholie, Francois Dabis, Emmanuel Bissagnene, IeDEA West Africa collaboration

**Affiliations:** 1 Service de Maladies Infectieuses et Tropicales (SMIT), CHU de Treichville, Abidjan, Côte d'Ivoire; 2 Université Bordeaux, ISPED, Centre INSERM U897- Epidémiologie-Biostatistique, Bordeaux, France; 3 INSERM, ISPED, Centre INSERM U897- Epidémiologie-Biostatistique, Bordeaux, France; 4 Centre de Prise en Charge des Personnes vivant avec le VIH, CNHU Cotonou, Bénin; 5 Service de cancérologie, CHU de Treichville, Abidjan, Côte d'Ivoire; 6 Laboratoire de cytologie et d'anatomopathologie, CHU de Cocody, Abidjan, Côte d'Ivoire; 7 Service de Médecine interne, CNHU, Cotonou, Bénin; Baylor College of Medicine, United States of America

## Abstract

**Background:**

Cancer is a growing co-morbidity among HIV-infected patients worldwide. With the scale-up of antiretroviral therapy (ART) in developing countries, cancer will contribute more and more to the HIV/AIDS disease burden. Our objective was to estimate the association between HIV infection and selected types of cancers among patients hospitalized for diagnosis or treatment of cancer in West Africa.

**Methods:**

A case-referent study was conducted in referral hospitals in Côte d’Ivoire and Benin. Each participating clinical ward enrolled all adult patients seeking care for a confirmed diagnosis of cancer and clinicians systematically proposed an HIV test. HIV prevalence was compared between AIDS-defining cancers and a subset of selected non-AIDS defining cancers to a referent group of non-AIDS defining cancers not reported in the literature to be positively or inversely associated with HIV. An unconditional logistic model was used to estimate odds ratios (OR) and their 95% confidence intervals (CI) of the risk of being HIV-infected for selected cancers sites compared to a referent group of other cancers.

**Results:**

The HIV overall prevalence was 12.3% (CI 10.3–14.4) among the 1,017 cancer cases included. A total of 442 patients constituted the referent group with an HIV prevalence of 4.7% (CI 2.8–6.7). In multivariate analysis, Kaposi sarcoma (OR 62.2 [CI 22.1–175.5]), non-Hodgkin lymphoma (4.0 [CI 2.0–8.0]), cervical cancer (OR 7.9 [CI 3.8–16.7]), anogenital cancer (OR 11.6 [CI 2.9–46.3]) and liver cancer (OR 2.7 [CI 1.1–7.7]) were all associated with HIV infection.

**Conclusions:**

In a time of expanding access to ART, AIDS-defining cancers remain highly associated with HIV infection. This is to our knowledge, the first study reporting a significant association between HIV infection and liver cancer in sub-Saharan Africa.

## Introduction

In industrialized countries, the advent of antiretroviral treatment (ART) has been marked by a substantial improvement in the duration of life of HIV-positive people together with the decline of AIDS defining illnesses [1], [2]. This quantitative and qualitative improvement in life expectancy has led many to consider that non-AIDS defining diseases including cancers would become soon a major cause of morbidity and mortality [3]. Infection with HIV is known to be associated with neoplasms such as Kaposi's sarcoma (KS), non-Hodgkin’s lymphoma (NHL) and Invasive Cervical Cancer (ICC) thus contributing to the definition of the AIDS stage [4]. Since the advent of ART, the incidence of these AIDS-defining cancers dropped in northern countries, with the clearest declines being experienced for KS and NHL [5]. Population-based record linkage studies in North America and Europe have identified in parallel several non AIDS-defining cancers as significantly associated with HIV infection [6]. With now more than six million patients on treatment, access to ART and subsequently survival of those treated have considerably increased over the last ten years in sub-Saharan Africa [7], [8]. Case-control studies and one record linkage study exploring the association between HIV infection and cancers have been conducted in Eastern and Southern Africa in a time of limited access to ART [9]–[12]. They have consistently reported a lower risk of AIDS-defining cancers in people living with HIV compared to pre-ART reports from industrialized countries [13]. Conversely, some non-AIDS defining cancers have been reported as associated with HIV in Africa such as Hodgkin’s disease, cancers of the anogenital organs and skin cancers [11]. In many West African countries, the association of cancer with HIV infection remains poorly understood. Furthermore, the distribution of cancers and exposure to carcinogens is different from other parts of sub-Saharan Africa. Indeed, some cancers considered as traditional because of linkage to endemic biological environmental agents are particularly represented in West Africa such as primary liver cancer related to hepatitis B virus infections. Others, such as KS related to Human Herpes Virus 8 infection seems to be less frequent compared to Central and Eastern Africa [14]. Our aim was to document the association between HIV infection and AIDS-defining cancers as well as some non-AIDS defining cancers in West Africa in the ART era.

## Materials and Methods

### Design and Study Population

A case-referent study was conducted in the three public referral hospitals of Abidjan, Côte d’Ivoire and in the referral hospital of Cotonou, Benin from October 2009 to October 2011. During the study period, clinical wards likely to manage patients with cancer were asked to include all patients attending with a diagnosis of cancer. In each participating ward, medical referents specifically designated for this study, contacted all adult patients (≥18 years old) seeking care with a suspected or confirmed diagnosis of cancer.

### Study Conduct

After obtaining patient’s written informed consent, pre-included subjects were administered a structured two-page questionnaire assessing socio-demographic characteristics (i.e. age, sex, lifetime number of sexual partners, place of living). The form was also designed to record from medical files clinical, biological, radiological and surgical data leading to the diagnosis of cancer. The medical files were regularly reviewed by medical referents and clinical monitors for diagnostic confirmation according to the latest available information. For diagnostic purposes, a cytological and/or histological examination, if medically indicated, was systematically proposed and financially supported by the research project. Confirmed cancer cases were classified according to their primary anatomical site of cancer (topography) and histological morphology when available using the WHO third edition of the International Classification of Diseases for Oncology (ICD-O-3) [15]. Medical referents were also responsible for proposing a systematic rapid HIV test (Determine®, Abbott Diagnostics) at the time of interview. They collected capillary blood by a finger prick test, after patient’s informed consent was obtained. In case of indeterminate result, the test was repeated once. In case of positive result or if still indeterminate after two attempts, a venous blood sample was collected for confirmation with a Genie2® (Bio-Rad, Marnes-La-Coquette, France) test allowing the identification of patients infected with HIV-1 alone, HIV-2 alone as well as dually infected patients. HIV testing was not repeated if the medical records indicated that the patient had previously been diagnosed HIV-positive. The study was approved by the national ethic committees of Benin and Côte d’Ivoire.

### Statistical Analysis

The frequency of HIV infection in selected types of cancer thought or known to be positively associated with HIV infection (i.e. KS, NHL, ICC, cancers of the anogenital organs, oral cavity, pharynx and larynx, Hodgkin lymphoma, squamous cell skin carcinoma, lung cancer, primary liver cancer and leukaemia) was compared to the frequency of HIV infection in a referent group of cancers. This referent group was chosen to be a mixture of cancer cases in order to compare to previously published case-control studies conducted in Southern Africa [10], [11]. Our referent group was thus constituted with cancers not reported in the current literature as positively or inversely associated with HIV infection (i.e. colorectal, breast, prostate, pancreas, oesophagus, endocrine system, ovary, endometrium, stomach, sarcomas other than KS, myelomas, kidney, bladder, biliary ducts & gallbladder, melanoma and mesothelioma). An unconditional logistic regression model was used to estimate the association by the computation of the odds ratios (ORs) with their 95% confidence intervals (95% CIs). In multivariate analysis, ORs were adjusted on age taken as continuous variable, gender (except for ICC) and lifetime number of sexual partners (<5 partners *versus* ≥5 partners). All statistical analyses were performed using SAS software, version 9.2 (SAS Institute Inc, Cary, NC, USA).

## Results

Of the 1,250 adult patients with a suspected or confirmed diagnosis of cancer, 233 (18.6%) were secondarily excluded: the HIV status could not be documented in 11 (0.9%) patients, 32 (2.6%) refused to participate after being provided with information about the risks and benefits of the study and 190 (15.2%) patients were excluded after a provisional diagnosis of cancer was dismissed. A total of 1,017 patients with a confirmed diagnosis of cancer and a documented HIV status were finally included in the present study. Their main characteristics according to HIV status are presented in table 1. The 128 patients identified as HIV-positive (92.2% HIV-1, 5.5% HIV-2 and 2.3% dually infected) were younger than the remaining 889 HIV-negative patients with median ages of 41 years [Interquartile range (IQR) 35–47] and 50 years [IQR 39–61], respectively (*p*<10^−4^).

**Table 1:** Characteristics of patients with a confirmed diagnosis of cancer according to HIV status (N = 1,017), IeDEA West Africa collaboration 2009–2011.

	HIV + (n = 128)	HIV – (n = 889)		Total (n = 1,017)
	n (%)	n (%)	*p*	n (%)
**Age**, median [IQR] (years)	41 [35–47]	50 [39–61]	<10^−4^	48 [38–60]
**Sex**			10^−4^	
Women	93 (72.7)	488 (54.9)		581 (57.1)
Men	35 (27.3)	401 (45.1)		436 (42.9)
**Place of living**			0.23	
Urban	51 (39.8)	306 (34.5)		357 (35.1)
Rural	77 (60.2)	583 (65.5)		660 (64.9)
**UTH**			0.03	
Treichville, Abidjan	64 (50.0)	373 (42.0)		437 (43.0)
Cocody, Abidjan	8 (6.2)	139 (15.6)		147 (14.4)
Yopougon, Abidjan	44 (34.4)	298 (33.5)		342 (33.6)
CNHU, Cotonou	12 (9.4)	79 (8.9)		91 (9.0)
**Formal education**			0.02	
Yes	95 (74.2)	602 (67.7)		697 (68.5)
No	29 (22.7)	278 (31.3)		307 (30.2)
Missing*	4 (3.1)	9 (1.0)		13 (1.3)
**Tobacco use** †			0.30	
Yes	10 (7.8)	96 (10.8)		106 (10.4)
No	118 (92.2)	793 (90.2)		911 (89.6)
**Alcohol use during last year**			0.96	
Yes	35 (27.3)	233 (26.2)		268 (26.3)
No	92 (71.9)	649 (73.0)		741 (72.9)
Missing*	1 (0.8)	7 (0.8)		8 (0.8)
**Personal history of cancer**			0.74	
No	126 (98.4)	860 (96.7)		986 (96.9)
Yes	1 (0.8)	12 (1.4)		13 (1.3)
Unknown	1 (0.8)	17 (1.9)		18 (1.8)
**Number of lifetime sexual partners**			0.02	
0 to 2	45 (35.2)	393 (44.2)		438 (43.1)
2 to 4	43 (33.6)	298 (33.5)		341 (33.5)
5 and over	36 (28.1)	154 (17.3)		190 (18.7)
Missing*	4 (3.1)	44 (4.9)		48 (4.7)
**Cancers with histology and/or cytology**	93 (72.7)	612 (68.8)	0.38	705 (69.3)
**Unknown HIV status prior to the study**	49 (38.3)	661 (74.3)	<10^−4^	710 (69.8)

Abbreviations: IQR Interquartile Range, UTH University Teaching Hospitals,

* missing values not documented during the data collection. Data completed for all included patients when not specified.

† Former or current smoker.

**Figure 1:**
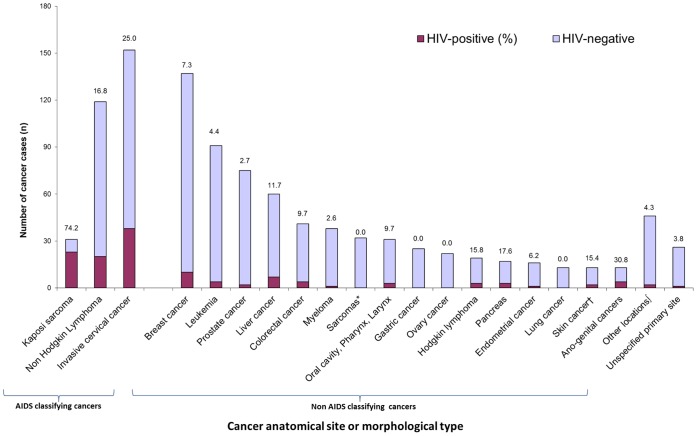
Distribution of patients according to their cancer primary site and HIV status in Côte d’Ivoire and Benin (N = 1,017), the IeDEA West Africa collaboration. 2009–2011. *Other than Kaposi sarcoma †Except melanoma ∫Other locations including esophagus (n = 10), endocrine tumors (N = 9), gallbladder & bile ducts (N = 8) and other minor cancer types (n = 20).

The three most common cancers were ICC (26.2%), breast cancer (23.1%) and NHL (9.6%) in women and prostate cancer (17.2%), NHL (14.4%) and liver cancer (10.5%) in men. The overall prevalence of HIV infection was estimated at 12.3% (95% CI 10.3–14.4). HIV infection was documented in 26.8% (95% CI 21.8–31.8) of the 302 patients with cancer considered as AIDS-defining and in 6.7% (95% CI 4.8–8.5) of the 689 patients with non-AIDS defining cancers. The estimated prevalence of HIV infection was 4.7% (95% CI 2.8–6.7) in the referent group of 442 patients with cancer types considered as not HIV-related including breast (n = 137), prostate (n = 75), colorectal (n = 41), myeloma (n = 38), sarcomas other than KS (n = 32), stomach (n = 25), ovary (n = 22), pancreas (n = 17), endometrium (n = 16), oesophagus (n = 10), endocrine tumours (n = 9), gallbladder and biliary ducts (n = 8), melanoma (n = 5), kidney (n = 4), bladder (n = 2) and mesothelioma (n = 1). The prevalence figures of HIV infection according to cancer types are displayed in figure 1.

**Figure 2:**
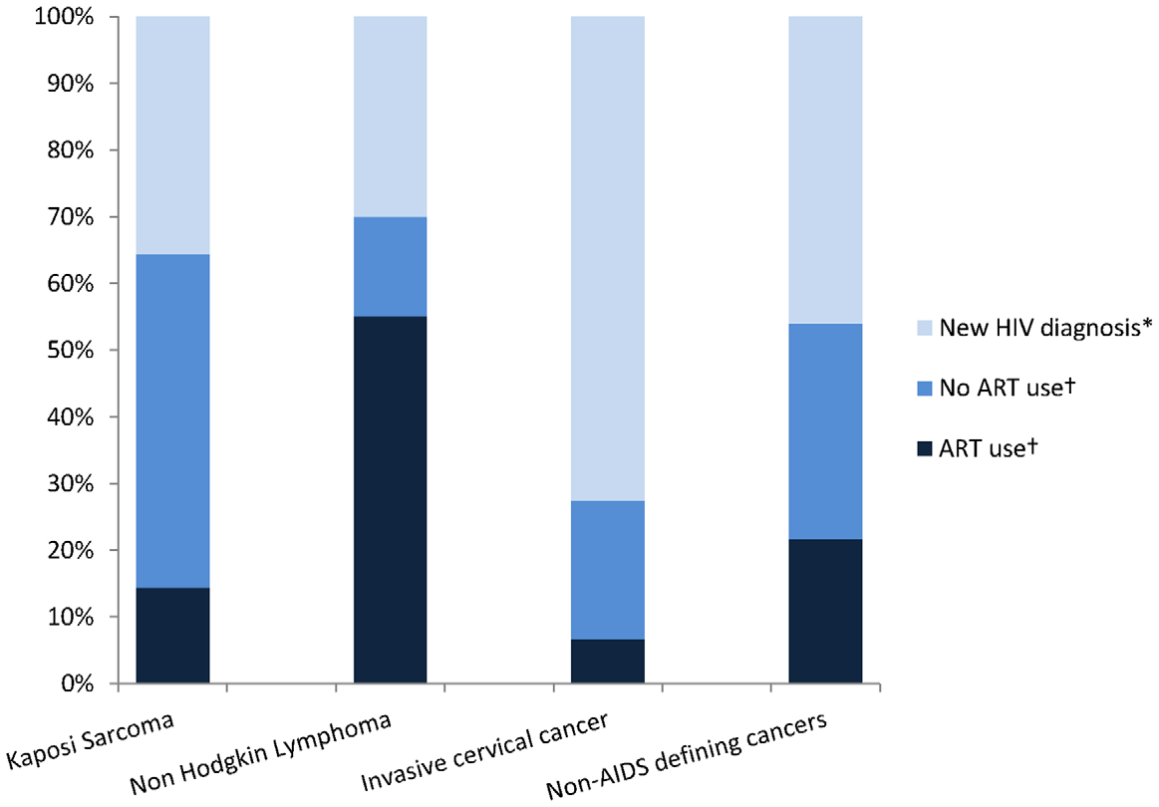
Distribution of ART use in HIV-positive patients according to the different AIDS and non-AIDS defining cancers **in Côte d’Ivoire and Benin, the IeDEA West Africa Collaboration, 2009–2011.** *Patients not known to be HIV-positive prior t the study conduction † Patients with a previously documented HIV infection.

Overall, 710 (69.8%) patients diagnosed with cancer had never been tested for HIV infection prior to the study and 49 of them (6.9%) were diagnosed HIV-positive in the course of the study. Among the 128 HIV-positive patients with cancer, 38.3% had never been tested for HIV infection prior to the study. In the 79 patients already known to be HIV-positive, 25 (31.6%) patients were already on ART, 26 (33.0%) were not currently on ART and for the remaining 28 (35.4%) patients; no information was available concerning ART exposure. Figure 2 summarises the distribution of ART use in all HIV-positive patients combined (i.e. those newly diagnosed, those already known to be HIV-infected) according to the different AIDS and non-AIDS defining cancers.

Of the 31 KS included, 29 (93.5%) had a histological confirmation and 23 (74.2%) were HIV-positive. In multivariate analysis, KS was significantly associated with HIV infection with an adjusted OR of 62.2 [95% CI 22.1–175.5] (Table 2).

**Table 2:** Association between selected cancers and HIV infection in Côte d’Ivoire and Benin, the IeDEA West Africa collaboration, 2009–2011.

Cancer site or morphological type (and ICD-O-3 code)		Univariate			Multivariate*		
	n/N** (%)	OR	95% CI	p	OR	95% CI	p
**Control group** †							
Total	21/442 (4.8)	1			1		
Women (for invasive cervical cancer)	12/257 (4.7)	1			1		
**AIDS defining cancers**							
Kaposi sarcoma (M9140)	**23/31 (74.2)**	**57.6**	**23.1–144.1**	**<10^−4^**	**62.2**	**22.1–175.5**	**<10^−4^**
Non Hodgkin lymphoma‡	**20/119 (16.8)**	**4.1**	**2.1–7.8**	**<10^−3^**	**4.0**	**2.0–8.0**	**<10^−2^**
Invasive cervical cancer (C53)	**38/152 (25.0)**	**6.8**	**3.4–13.5**	**<10^−4^**	**7.9**	**3.8–16.7**	**<10^−4^**
**Non AIDS defining cancers**							
Anogenital organs (C21, C51, C52, C60)	**4/13 (30.8)**	**8.9**	**2.5–31.3**	**<10^−3^**	**11.6**	**2.9–46.3**	**<10^−3^**
Oral cavity, pharynx, larynx (C00– C10, C32)	3/31 (9.7)	2.1	0.6–7.6	0.23	1.0	0.2–4.9	0.99
Squamous cell skin cancers (C44, M8050–8082)	2/13 (15.4)	3.6	0.8–17.5	0.10	3.4	0.6–18.3	0.14
Hodgkin lymphoma‡	3/19 (15.8)	3.8	1.0–13.9	0.04	3.0	0.7–13.3	0.15
Primary liver cancers (C22)	**7/60 (11.7)**	**2.6**	**1.1–6.5**	**0.03**	**2.7**	**1.1–7.7**	**0.04**
Leukaemia (C42, M9800–9891)	4/91 (4.4)	0.9	0.3–2.7	0.88	0.8	0.2–2.4	0.65

* Adjusted on age taken as a continuous variable, gender (except for invasive cervical cancer) and lifetime number of sexual partners (<5 *versus* ≥5).

** n/N: number of HIV+ patients/number of patients with cancer.

† Control group of cancers not known to be related with HIV infection from the existing literature: prostate, breast, colon/rectum, oesophagus, stomach, pancreas, endometrium, ovary, endocrine, sarcomas other than Kaposi sarcoma.

‡ See Table S1 for morphological types.

Abbreviations: OR Odd Ratio, CI Confidence Interval.

In the 119 patients diagnosed with an NHL, 20 (16.8%) were HIV-positive, with a significantly lower median age at diagnosis compared to the HIV-negative ones, 41.5 years [IQR 32.5–51.0] *versus* 52.0 years [IQR 34.0–63.0] respectively, (*p* = 0.02). The diagnosis of NHL was documented by immunohistochemistry in 22.7% of cases or otherwise by cytology and/or histology. Morphological types of NHL according to HIV status are presented as Table S1. Of the 119 NHL included, 81 (68.1%) had a documented morphological type available. Mature B-cell NHL represented 86.4% of NHL with specified morphological type. Marginal zone B-cell (11.8%), diffuse large B-cell (10.9%), small B-cell lymphocytic (8.4%) and Burkitt lymphoma (8.4%) were the four most commonly reported types of NHL irrespective of HIV status. Among the 20 HIV-positive patients with a diagnosis of NHL, Burkitt lymphoma was the most frequent morphological type with four (20.0%) cases reported versus six (6.1%) in HIV-negative patients (*p* = 0.08). In multivariate analysis, NHL was significantly associated with HIV infection with an adjusted OR of 4.0 [95% CI 2.0–8.0].

The prevalence of HIV infection in the 152 women with ICC was 25.0%. A histological confirmation was reported in 131 (85.5%) cases, including 117 squamous cell carcinomas and 14 adenocarcinomas. The median age of these series was 43.5 years [IQR 36.5–48.0] in HIV-positive women and 51.0 years [IQR 43.0–60.0] in HIV-negative women (p<10^−4^). Clinical stage at diagnosis according to the International Federation of Gynaecology and Obstetrics (FIGO) classification was documented in 104 (68.4%) women. Extended ICC at diagnosis (stage 3 or 4) was reported in 24 (80%) HIV-positive and 53 (71.6%) HIV-negative women with documented FIGO staging (*p* = 0.37). In multivariate analysis, ICC was significantly associated with HIV infection with an adjusted OR of 7.9 [95% CI 3.8–16.7].

Of the 13 cancers of the anogenital organs reported, 11 (84.6%) had histological confirmation. Six (46.2%) were vulvovaginal cancers and seven (53.8%) were cancers of the anus of which five (71.4%) occurred in women. Overall, cancers of the anogenital organs were associated with HIV infection (adjusted OR = 11.6 [95% CI 2.9–46.3]). The 13 cases of squamous cell skin carcinoma and the 19 cases of Hodgkin lymphoma were all confirmed by pathological examination. The prevalence of HIV infection was 15.4% in squamous cell skin carcinoma and 15.8% in Hodgkin lymphoma. However, the association between HIV infection and these two cancers was not statistically significant with adjusted ORs of 3.4 [95% CI 0.6–18.3] and 3.0 [95% CI 0.7–13.3], respectively. A total of 14 cancers of oral cavity, 12 cancers of the larynx and five cancers of the pharynx were diagnosed. Of these 31 cancers, 26 (67.7%) were confirmed by histological examination of which 21 (80.8%) were squamous cell carcinoma. Altogether, cancers of the oral cavity, larynx and pharynx were not associated with HIV infection with an OR of 1.0 [95% CI 0.2–4.9]. When considering all non AIDS defining cancers suspected or known to be also HPV-related (i.e. cancers of the anogenital organs, squamous cell skin carcinoma, head and neck cancers) HIV infection was diagnosed in 9/57 (15.8%) patients, yielding an adjusted OR of 3.1 [95% CI 1.3–7.8]. A total of 13 bronco-pulmonary cancers were documented and none of them were found to be HIV-positive. Primary liver cancer was diagnosed in 60 patients and seven (11.7%) were HIV-positive, for an adjusted OR of 2.7 [95% CI 1.1–7.7]. The median age of patients with primary liver cancer was 32 years [IQR 31–44] in HIV-positive patients and 49 years [IQR 44–59] in HIV-negative patients (*p*<10^−2^). Leukaemia was not associated with HIV infection (adjusted OR = 0.8 [95% CI 0.2–2.4]). HIV infection was documented in three (13.0%) of the 23 acute leukaemia, one (2.0%) of the 51 chronic myeloid leukaemia and none of the 17 chronic lymphoid leukaemia.

## Discussion

In a time of expanding access to ART, AIDS defining as well as some non-AIDS defining cancers were significantly associated with HIV infection in this multi-country survey in West Africa. KS and NHL were associated with HIV infection, with a similar magnitude of association than in previous case-control studies from sub-Saharan Africa performed prior to the roll out of ART [10], [11], [16]. In Côte d’Ivoire, access to ART has started in 2002 and is free of charge since 2007 [17]. Three years later, KS and NHL were still highly associated with HIV infection according to our findings. Reports from population-based record linkage studies in industrialized countries have constantly documented a prompt decline in the occurrence of KS and NHL since the advent of ART [5], [18]. Engels *et al* have compared standardized incidence ratios (SIRs) of KS and NHL between the 1991–1995 and the 1996–2002 calendar periods in the USA. The SIRs decreased from 2,800 [95% CI 2,300–3,500] to 790 [95% CI 640–980] for KS and from 9.8 [95% CI 7.7–12] to 6.5 [95% CI 5.4–7.7] for NHL. In the present study, the 60-fold increased risk of being HIV-positive when presenting with KS as well as the four-fold increased risk of being HIV-positive when presenting with NHL are quite similar to figures reported in previous case-control studies conducted in Africa more than 10 years ago. At least two explanations can be formulated: first, it might be too early to observe a significant decrease in the occurrence of NHL and KS considering the incomplete coverage of ART and the high HIV prevalence with a large proportion of HIV-positive people in need for a treatment remaining undiagnosed or not yet in care with ART [7]. This first hypothesis is supported by the low frequency of ART use among HIV-positive patients reported in the present study. Second, the association between KS, NHL and HIV infection has been constantly reported to be lower in Africa than in industrialized countries and might have shaded the potential impact of ART [13].

NHL presented with a wide variety of histological subtypes in our series, as already documented elsewhere in sub-Saharan Africa [19]. Burkitt lymphoma was common within the NHL series with a well-documented morphological type, especially in HIV-positive patients, consistent with prior reports from South Africa [11], [20]. Unlike other NHL subtypes such as diffuse large B-cell lymphoma or primary brain lymphoma, Burkitt lymphoma does not seem to be influenced by cellular immunity [21], [22]. The high frequency of Burkitt lymphoma might contribute to the lower-than-expected association between NHL and HIV report in sub-Saharan African studies, together with a competitive mortality due to communicable diseases such as tuberculosis. In addition, the high frequency of Burkitt lymphoma observed in HIV-positive patients might also challenge the potential positive impact of ART on NHL in the coming years.

According to the international agency research on cancer (IARC) latest estimates in sub-Saharan Africa, ICC is the leading cause of cancer in women regardless of HIV infection [23]. Several genital human papillomavirus (HPV) types have been identified as the necessary cofactor for ICC and its histological precursor and classified as carcinogenic according to the IARC classification [24]. HIV infection and its associated immunosuppression have been repeatedly linked with carcinogenic HPV infection in sub-Saharan Africa [25]–[27]. However, previous case-control studies conducted in other parts of Africa found no or weak association between HIV and ICC [13]. In our present study, ICC was strongly associated with HIV infection. A previous case-control study assessing the relationship between HIV infection and ICC was conducted in Côte d’Ivoire from April 1997 to October 1999. The prevalence of HIV infection was 16.7% among 132 patients with ICC compared to 8.3% among 120 patients in the control group, giving an adjusted OR of 3.4 [95% CI 1.4–8.3] [28]. The HIV prevalence reported in their control group was consistent with the HIV prevalence in adult women in Côte d’Ivoire ten years earlier. Conversely, we report today a higher frequency of HIV infection in women diagnosed with ICC. As the majority of these women with ICC was unaware of their HIV status and thus untreated, the high frequency of HIV infection we report is not likely to be related to a prolonged life expectancy while on ART. According to ICC natural history, progression to invasive stages takes years to decades to occur. A latency period is thus expected between the HIV epidemic that peaked during the late 90′s in most countries in West Africa and its impact on the occurrence of ICC. While HIV prevalence figures in the general population of adult women in Côte d’Ivoire and Benin have fallen during the last ten years, women with ICC continue to present with a high and sustained HIV prevalence explaining the particularly high association between ICC and HIV infection reported in our study. However, comparing cancer rates by calendar period from case-referent studies conducted in different settings with various methodologies does not allow any definite conclusion on trends in cancer incidence according to HIV status. Assessing the true impact of HIV infection and exposure to ART on cancer trends such as ICC in sub-Saharan Africa will ultimately need HIV and cancer registries matched studies. Cancer of the anogenital organs which share an important risk factor with ICC, namely HPV infection, were highly associated with HIV infection, a finding consistent with a previous report from South Africa [11]. Primary liver cancer has been reported as associated with HIV infection in industrialized countries. Indeed, a meta-analysis of the incidence of non-AIDS cancers according to HIV status from 18 studies reported an increased standardized incidence ratio of 5.6 [95% CI 4.0–7.7] for primary liver cancer [6]. To our knowledge, this is the first study reporting an association between primary liver cancer and HIV in sub-Saharan Africa. Immunosuppression induced by HIV infection might play a direct role in the occurrence of this cancer as suggested by a meta-analysis reporting a higher incidence of liver cancer in both HIV-infected patients as well as transplant patients compared to the general population [29]. In industrialized countries, the prevalence of hepatitis B virus (HBV) and hepatitis C virus (HCV), infectious agents leading to liver cancer, are higher among HIV-infected patients compared to the general population as they share the same routes of transmission (i.e. through blood contamination or sexual relation). In sub-Saharan Africa, especially the West Africa part, HBV infection is highly prevalent and usually acquired during childhood whereas this occurs later in life in industrialized countries. These different transmission dynamics between the two types of settings might directly impact on the risk of HBV infection according to HIV status. Indeed, several studies reported similar seroprevalence figures of HBV infection in HIV-positive adults compared to HIV-negative ones [30], [31]. However, the exact impact of HIV infection on HBV infection and the risk of subsequent liver cancer remain to be explored in sub-Saharan Africa. Dedicated case-control studies taking into account the main known risk factors for primary liver cancer (HBV, HCV, as well as aflatoxin exposure) are needed to explore more accurately this association.

### Limitations

Our study population might not reflect the exact distribution of cancers occurring in these two countries of West Africa. Since there were no functional population-based cancer registries in the catchment area of the four referral hospitals and no hospital-based cancer registry during the study period we were not able to ensure that all cancer cases were screened during the study period. Additionally, we disregarded patients with cancers attending exclusively the private health sector. Thus, the present report does not pretend to be fully representative of the distribution of cancers in Côte d’Ivoire and Benin. However, the pattern of cancers reported was quite similar to the distribution of cancers reported in the past by the former cancer registry of Côte d’Ivoire and from most recent estimated data reported by the Globocan program for Côte d’Ivoire and Benin [14], [23]. Based on multiple sources of information (referral hospitals, government and private hospitals, health centers and private clinics), Echimane *et al* had indeed registered 1,871 cancers in the urban area of Abidjan during the 1995–97 period. Prostate cancer (15.0%), liver cancer (14.6%) and NHL (10.0%) were the three most frequent malignancies registered in men and breast cancer (25.2%), ICC (23.6%) and NHL (7.2%) the three most frequent malignancies in women, concordant with the cancer distribution reported in our study 15 years later. One could argue that the choice of a referent group constituted only of cancer patients might not be appropriate for HIV prevalence comparisons. A previous South African case-control study measuring the association between HIV infection and selected type of cancers has both recruited their control group among cancer patients and patients consulting for cardiovascular disease [11]. They found no significant difference in the prevalence of HIV infection among these two groups of control subjects, after adjustment for age, year of diagnosis and gender. However, we acknowledge that potential biases might have been introduced by using cancer cases as a referent group. Indeed, the HIV prevalence of our hospital-based referent group was higher than the estimated HIV prevalence of 3.4% [95% CI 3.1–3.9] reported in the adult (15–49 year old) general population of Côte d’Ivoire in 2009, thus potentially underestimating ORs estimates [32]. However, differences in the age distribution and other characteristics related to hospital subjects limit comparisons between our hospital-based group to the general adult population. The limited number of cancer cases in specific anatomic localizations prevented a precise assessment of the association between these cancers and HIV infection. The extension of such a case-referent study to other referral hospital from other countries with generalized HIV epidemic in West Africa will provide a unique opportunity to explore rarer cancers types suspected to be HIV-related. Finally, the cross-sectional nature of the study prevents from drawing any causal relation between HIV infection and cancer. In a context where the collection of prospective cancer cases is particularly challenging, a cross-sectional approach that could be repeated over time using the same methodology might however provide useful information on cancer trends, paving the path for the development and strengthening of cancer registries.

### Conclusion

In a time of expanding access to ART, AIDS-defining cancers remain highly associated with HIV infection in West Africa. Some non-AIDS defining cancers such as cancer of anogenital organs or primary liver cancer are also associated with HIV infection. It is a public health commitment for oncologists and other practitioners in charge of cancer patients to offer systematic screening of HIV infection. Indeed, this practice was not routine prior to the conduct of this study with nearly half of the HIV-positive patients newly diagnosed as a direct consequence of the study. The low proportion of refusals we observed indicates that is now acceptable and workable to perform provider-initiated HIV testing in patients suspected or diagnosed with cancer.

## Supporting Information

Table S1 Morphological types of lymphomas (N = 119) according to HIV status, the IeDEA West Africa collaboration, 2009–2011. *Morphological subtypes according the international classification of disease in oncology third edition (ICD-O3). Abbreviations: NHL: Non-Hodgkin Lymphoma, NOS None Otherwise Specified.
(DOCX)

Appendix S1 The International epidemiological Database to Evaluate AIDS (IeDEA) collaboration in West Africa. *Member of the IeDEA West Africa Technical Committee.
(DOCX)
