# Supplementary material for: The Spectrum of Cancers in West Africa: Associations with Human Immunodeficiency Virus
Source: PLoS One. 2012 Oct 29;7(10):e48108. doi: 10.1371/journal.pone.0048108 (PMC3483170; doi:10.1371/journal.pone.0048108)
Supplement: Table S1 — Morphological types of lymphomas (N = 119) according to HIV status, the IeDEA West Africa collaboration, 2009–2011. *Morphological subtypes according the international classification of disease in oncology third edition (ICD-O3). Abbreviations: NHL: Non-Hodgkin Lymphoma, NOS None Otherwise Specified. (DOCX) [file pone.0048108.s001.docx]

**Table S2. Morphological types of lymphomas (N=119) according to HIV status, the IeDEA West Africa collaboration, 2009-2011**

|  | Morphology code | HIV + | HIV – |
| --- | --- | --- | --- |
|  | ICD-O-3* | N (%) | N (%) |
| **Non-Hodgkin lymphoma** |  | 20 (16.8) | 99 (83.2) |
| NHL, NOS | 9591 | 5 (25.0) | 33 (33.3) |
| **Mature B-cell NHL** |  |  |  |
| Small B-cell lymphocytic, NOS | 9670 | 2 (10.0) | 8 (8.1) |
| Lymphoplasmacytic lymphoma | 9671 | 1 (5.0) | 4 (4.0) |
| Centrocytic lymphoma | 9673 | 1 (5.0) | 5 (5.0) |
| Mixed small and large cell, diffuse lymphoma | 9675 | 3 (15.0) | 5 (5.0) |
| Large B-cell, diffuse lymphoma, NOS | 9680 | 0 (0.0) | 13 (13.1) |
| Immunoblastic lymphoma, NOS | 9684 | 0 (0.0) | 1 (1.0) |
| Burkitt lymphoma | 9687 | 4 (20.0) | 6 (6.1) |
| Follicular lymphoma, NOS | 9690 | 0 (0.0) | 1 (1.0) |
| Follicular lymphoma of grade 3 | 9698 | 0 (0.0) | 2 (2.0) |
| Marginal zone B-cell lymphoma, NOS | 9699 | 0 (0.0) | 14 (14.1) |
| **Mature T-cell NHL** |  |  |  |
| Mature T-cell lymphoma, NOS | 9702 | 1 (5.0) | 3 (3.0) |
| Subcutaneous panniculitis-like T-cell lymphoma | 9708 | 2 (10.0) | 0 (0.0) |
| Anaplastic large-cell lymphoma | 9714 | 0 (0.0) | 2 (2.0) |
| **Precursor-cell NHL** |  |  |  |
| Lymphoblastic lymphoma | 9727 | 1 (5.0) | 2 (2.0) |
| **Hodgkin lymphoma** |  | 3 (15.8) | 16 (84.2) |
| Hodgkin lymphoma, NOS | 9650 | 1 (33.3) | 6 (37.2) |
| Lymphocyte-rich classical Hodgkin lymphoma | 9651 | 0 (0.0) | 1 (6.2) |
| Mixed cellularity classical Hodgkin lymphoma | 9652 | 0 (0.0) | 2 (12.4) |
| Hodgkin granuloma | 9661 | 0 (0.0) | 1 (6.2) |
| Hodgkin sarcoma | 9662 | 0 (0.0) | 1 (6.2) |
| Nodular sclerosis, NOS | 9663 | 0 (0.0) | 1 (6.2) |
| Nodular sclerosis, lymphocytic predominance | 9665 | 1 (33.3) | 1 (6.2) |
| Nodular sclerosis, grade 2 | 9667 | 1 (33.3) | 3 (18.6) |

* Morphological subtypes according the international classification of disease in oncology third edition (ICD-O3)

Abbreviations: NHL: Non-Hodgkin Lymphoma, NOS None Otherwise Specified
