# Supplementary material for: The Spectrum of Cancers in West Africa: Associations with Human Immunodeficiency Virus
Source: PLoS One. 2012 Oct 29;7(10):e48108. doi: 10.1371/journal.pone.0048108 (PMC3483170; doi:10.1371/journal.pone.0048108)
Supplement: Appendix S1 — The International epidemiological Database to Evaluate AIDS (IeDEA) collaboration in West Africa. *Member of the IeDEA West Africa Technical Committee. (DOCX) [file pone.0048108.s002.docx]

**Appendix S1. The International epidemiological Database to Evaluate AIDS (IeDEA) in West Africa collaboration**

- Primary Investigators: Pr François Dabis* (INSERM U897, ISPED, Bordeaux, France), Pr Emmanuel Bissagnene* (SMIT, CHU de Treichville, Abidjan, Côte d’Ivoire)

- Clinical Investigators by country and alphabetical order: Jocelyn Akakpo, Alain Azondékon, Jules Bashi, Sagbo Gratien, Sikiratou Koumakpaï, Marcel D. Zannou* (Benin); Ye Diarra, Eric-Arnaud Diendere, Joseph Drabo*, Fla Koueta (Burkina Faso); Edmond Aka-Addi, Clarisse Amani-Bosse, Franck-Olivier Ba-Gomis, François Eboua-Tanoh, Serge-Paul Eholie*, Calixte Guehi, Kouakou Kouadio, Serge-Olivier Koulé, Eugène Messou, Albert Minga, Aristophane Tanon, Marguerite Timité-Konan, Pety Touré, (Côte d’Ivoire); Kevin Peterson* (Gambia); Bamenla Goka, Lorna Renner* (Ghana); Hadizatou Coulibaly, Fatoumata Dicko, Moussa Maiga*, Daouda Minta, Mariam Sylla, Hamar Alassane Traoré; Man Charurat* (Nigeria); Bernard Diop, Fatou Ly Ndiaye, Papa Salif Sow, Haby Signaté Sy*, Judicaël Tine (Senegal)

- Epidemiology and Statistical Unit (INSERM U897, ISPED, Université Victor Segalen, Bordeaux, France): Eric Balestre, Didier K. Ekouévi*, Antoine Jaquet*, Valériane Leroy*, Charlotte Lewden*, Karen Malateste, Annie J. Sasco*, Rodolphe Thiebaut

- Data Management Unit (PACCI, CHU Treichville, Abidjan, Côte d’Ivoire): Gérard Allou, Jean Claude Azani, Patrick Coffie

- Administration: Alexandra Doring and Elodie Rabourdin (ISPED), Hughes Djétouan, Bertin Kouadio and Adrienne Kouakou (PACCI)

- Adult clinical centers by city and country:

Abidjan, Côte d’Ivoire: Service de Maladies Infectieuses et Tropicales (SMIT), CHU de Treichville, Unité de Soins Ambulatoires et de Conseil (USAC), Centre Médical de Suivi de Donneurs de Sang/CNTS/PRIMO-CI, ACONDA-MTCT-Plus, ACONDA-CePReF, Centre Intégré de Recherche Bioclinique d’Abidjan (CIRBA).

Abuja, Nigeria: University of Abuja Teaching Hospital (UATH). Bamako, Mali: Service d’Hépato-Gastro-Entérologie, Hôpital Gabriel Touré, Centre de Prise en Charge des Personnes vivant avec le VIH, Hôpital du Point G. Dakar, Sénégal: Service des Maladies Infectieuses, CHU de FANN/ ISAARV. Banjul, Gambia: Fajara Cohort. Benin City, Nigeria: University of Benin Teaching Hospital (UBTH) Cotonou, Benin: Service de Médecine Interne, CNHU Hubert Maga. Ouagadougou, Burkina-Faso: Service de Médecine Interne, CHU Yalgado

- Pediatric clinical centers by city and country:

Abidjan, Côte d’Ivoire: ACONDA-CEPREF, ACONDA-MTCT-Plus, CHU de Yopougon, Centre Intégré de Recherche Bioclinique d’Abidjan (CIRBA). Accra, Ghana: Korle Bu Teaching Hospital. Bamako, Mali: Hôpital Gabriel Touré. Cotonou, Benin: Centre National Hospitalo-Universitaire Hubert Maga, Hôpital d’Instruction des Armées. Dakar, Senegal: Hôpital d’Enfants Albert-Royer. Fajara, Gambia: Medical Research Council. Ouagadougou, Burkina-Faso: Centre Hospitalier Charles de Gaulle.

* Member of the IeDEA West Africa Technical Committee
